# M. Velpeau on the Hymen

**Published:** 1842-05-28

**Authors:** 


*The Hymen.*—M. Velpeau states in his lectures on the diseases of the uterus, that the tubercles which result from the rupture of the hymen, instead of becoming atrophied, as is generally the case, occasionally enlarge and are the seat of much irritation, so as to be mistaken for polypi or the effect of venereal disease. Astringent lotions, or excision, will effect a speedy and perfect cure.—*Provincial Medical and Surgical Journal*, from *Examinateur Médical.*

